# A Plea for Plaster of Paris in the Treatment of Fracture

**Published:** 1886-01-20

**Authors:** L. Edwards Borcheim

**Affiliations:** Atlanta, Ga.


### A PLEA FOR PLASTER OF PARIS IN THE TREATMENT OF FRACTURE.

BY L. EDWARDS BORCHEIM, M. D., ATLANTA, GA.

That at this late day a defence of the immediate use of the permanent bandage in the treatment of fracture should be necessary will, to the majority of surgeons, be in the nature of a revelation ; yet, in the annual report of the Georgia Medical Association, and copied in the SOUTHERN MEDICAL RECORD, I read a paper by W. H. Elliot, of Savannah, in which he is made to say : "I must here record my protest against the practice of putting a bandage around a broken limb before inflammation has taken place, except as a temporary expedient for the removal of a patient," etc. ; then he goes on to describe my late esteemed colleague's (Dr. Little's) method, which had been put in practice by that distinguished Surgeon since 1860, and published and popularized by him in a paper read before the American Medical Association in 1867 (see Transactions). While practicing in New York City, as house physician to the Mount Sinai Hospital, the treatment of fractures was an every-day occurrence. Fractures of the upper extremity were always permanently dressed with starch, plaster of Paris, or silicate of soda bandages, and the patient dismissed and treated as an outdoor case. Those of the lower extremity were always dressed similarly, when seen before the commencement of the inflammatory reaction.

C. Heath, in his Minor Surgery, says : "The time chosen for putting up fractures varies, some surgeons applying splints, etc., immediately, others preferring to wait until all swelling has subsided; but this only applies to fractures of the lower extremity, those of the upper being dressed at once and treated, for the most part, as out patients."

Erichsen (Science and Art of Surgery) says: "During many years I have employed Seutin's plan (starched bandage) in several hundred cases of fracture, *immediately* after reduction of fracture—even in fractures of the thigh—in simple as well as compound fracture."

Neudorfer lays great stress on the fact that it should be applied immediately; as does Esmarch, of Kiel, Volkmann, of Halle, and Packard, of Philadelphia. Need I add the result of personal experience? I think not; but simply recommend that whenever and wherever possible, the surgeon should at once reduce the fracture and apply a permanent dressing, thereby saving the patient's time, strength and vigor, and gaining for himself honor and credit.

